# Social Insurance Literacy of Dutch Workers Receiving Disability Benefits and its Associations with Socio-Economic Characteristics

**DOI:** 10.1007/s10926-021-10018-3

**Published:** 2022-01-05

**Authors:** M. D. Boonstra, F. I. Abma, L. Wilming, C. Ståhl, E. Karlsson, S. Brouwer

**Affiliations:** 1 Department of Health Sciences, University of Groningen, University Medical Center Groningen, Hanzeplein 1, Groningen, Groningen The Netherlands; 2 Research Center for Insurance Medicine, Amsterdam, The Netherlands; 3 Department of Behavioural Sciences and Learning, Linköping University, Linköping, Sweden; 4 HELIX Competence Center, Linköping University, Linköping, Sweden; 5 Department of Health, Medicine and Caring Sciences, Linköping University, Linköping, Sweden

**Keywords:** Social security, Literacy, Comprehension, Socio-economic background, Cross-sectional studies

## Abstract

*Purpose* This study explores the concept social insurance literacy (SIL) and corresponding questionnaire (SILQ) among workers receiving disability benefits and the comprehensibility of the social security institute (SSI), and examines associations with socio-economic characteristics. *Methods* 1753 panel members of the Dutch SSI were approached to complete the SILQ-NL37. This measure was based on the original SILQ. The SILQ-NL37 contains domains for obtaining, understanding and acting upon information for both individual SIL and system comprehensibility. A higher score means better SIL or comprehensibility. Data on age, gender, education, living situation, Dutch skills and time receiving disability benefits were also collected. With k-means clustering, groups with adequate and limited SIL were created. Associations with socio-economic characteristics were examined with independent t-tests and linear regression analyses for both the total scores and within domain scores. *Cronbach α* and *Spearman rho’s* indicated measurement properties were good to acceptable for the SILQ-NL37. *Results* Thirty-five percent of the 567 participants were in the group with limited SIL. Higher individual SILQ-NL37 scores were associated with having a partner (p = 0.018) and northeastern living region (p = 0.031). Higher scores for obtaining (p = 0.041) and understanding (p = 0.049) information were associated with female sex, and for acting on information with younger age (p = 0.020). People with limited Dutch skills (p = 0.063) and a partner (p = 0.085) rated system comprehensibility higher. *Conclusions* According to the SILQ-NL37 scores, about 35% of the panel members have limited ability to obtain, understand and act upon social insurance systems information. Limited SIL is associated with several socio-economic factors. Future researches should study the concept in a more representative sample, and in different countries and social insurance contexts.

**Supplementary Information:**

The online version contains supplementary material available at 10.1007/s10926-021-10018-3.

## Introduction

When applying for a work disability benefit within social insurance systems, applicants must possess knowledge of the requirements and have the skills to correctly follow the application process. For example, applicants need to fill in multiple forms, provide information on their illness, meet with physicians and social security professionals, and draft and reflect upon action plans for re-integration [1]. The burden of processes within social insurance systems is associated with negative health effects, feelings of mistreatment, perceived system injustice and client’ dissatisfaction [2–4]. Benefit decisions may be influenced by the individuals’ abilities to successfully complete all processes, but also by the systems’ capacity to meet the needs of individuals who lack these abilities [5].

The concept social insurance literacy (SIL) was recently developed to identify factors that influence the people’s contact with social insurance systems and underlines the role of both the individual and the system. SIL is defined as the extent to which individuals can obtain, understand and act on information in a social insurance system, related to the comprehensibility of the information provided by the system [6]*.* The above definition was established through a scoping literature review and a workshop with an international expert panel with expertise in sociology, social medicine, law, rehabilitation, social insurance, epidemiology, occupational therapy, and public health.

SIL has its roots in theories in sociology, social medicine and public health, and is derived from health literacy (HL) and other related concepts such as financial literacy [6]. HL problems are associated with multiple negative societal outcomes, such as higher mortality, worse health and health inequalities [7, 8]. According to theories, these worse outcomes result from the impact of HL on other factors, such as disease knowledge, self-care behaviors, communication and access to care [9, 10]. On an individual level, low socio-economic status is a predictor of limited HL and financial literacy [11, 12]. SIL is likely to share similarities with HL with an impact on different outcomes and associations with socio-economic factors, but this is still unknown.

There is evidence that people applying for disability benefits consider the application processes as burdensome [2–4], not client centered, and that information does not meet their needs [13, 14]. Also in the Netherlands, the process to receive a disability benefit encompasses many steps. The Work and Income Act (WIA) allows employees to apply for a disability benefit after two years of sick leave [15]. They apply for a disability benefit from the Dutch social security institute (SSI) for Employee Benefits Schemes (in Dutch: UWV). Based on application forms and a medical disability assessment by an insurance physician and assessing of earning capacity by a labor expert, people are granted a full and permanent work disability, a non-permanent but full work disability, or a permanent and partial work disability benefit. Yearly, 5% of the applications are rejected, but 4.6% of those people recovered or received another type of benefit.

There is some evidence on the complexity and burden of social insurance processes. Although, the extent to which people have the abilities to meet the requirements of social insurance systems has not yet been extensively studied. It is also unclear to what extent these systems are accessible and comprehensible for the applicants. The Social Insurance Literacy Questionnaire (SILQ) was therefore recently developed by an international group of researchers [6] to further investigate the needed individual’s abilities to obtain, understand, and act on information of those systems within three domains: contacts and communication, system navigation and decisions and appeals. Additionally, it measures the comprehensibility of the system, i.e. the systems’ ability to provide accessible, understandable, and transparent information. Results on the development and validation of the original SILQ are forthcoming.

Against this background, the aim of this study was to explore SIL among individuals who were granted work disability benefits and the comprehensibility of the Dutch SSI, the governing authority responsible for employee insurances. Additionally, the associations between individual SIL, comprehensibility and socio-economic characteristics respectively were examined.

## Methods

### Study Design

In this cross-sectional study, members of an online panel of claimants of the Dutch SSI, called the UWV, were approached to fill in a shortened and culturally adapted version of the SILQ (SILQ-NL37) and additional questions on socio-economic characteristics. The Central Medical Ethical committee of the University Medical Center Groningen (UMCG) reviewed and approved this study (Number: 201900597).

### Participants and Data Collection

1753 members of an online panel of the Dutch SSI were invited to participate in this study. The SSI frequently uses the online panel to ask for feedback on quality of service, policy changes and for scientific research. Prior to our study, these members pro-actively registered for the panel and provided informed consent to be approached for these purposes regularly. Data on age, gender, education, postal code and living situation were collected in an online questionnaire system.

For our study, members were eligible for inclusion when they were 1) client of the SSI, 2) received a work disability benefit after the two-year waiting period of sick leave. This benefit falls under the regulations of the WIA [15]. The WIA distinguishes between people who are fully unable to work (WIA-IVA), and people who have residual working capacity (WIA-WGA). We specifically selected the last group in our study sample, which has the largest total population and is the most diverse in background characteristics (i.e. age, education) [16].

The questionnaire was programmed in the questionnaire system, and for all items it was obliged to select an answer, preventing missing answers. In November 2019, the SSI send an e-mail explaining the study and with a link to the questionnaire to eligible panel members. Based on the explanatory information, panel members chose if they wanted to participate or not. After one week a reminder was sent. Two weeks after the first e-mail, data were exported in an SPSS-file.

### Measures

*Social Insurance Literacy Questionnaire*—to explore the SIL of individuals and the comprehensibility of the system, we administered the SILQ-NL37. This measure is derived from the original version of the SILQ, developed in 2019[6], which contained 45 items on individual abilities and 24 items on system comprehensibility. For the abilities, the SILQ focuses on different abilities (i.e. obtaining, understanding and acting upon information) within different domains (i.e. contacts and communication, navigating the system, and decisions and appeals). Items regarding system comprehensibility are organized within the same domains.

At an early stage, the original SILQ was evaluated with regards to content validity. This evaluation was performed through a Delphi-study. An international expert panel reviewed the items of the SILQ in relation to the definition and domains of SIL. Revisions of the SILQ were made until consensus was reached upon the relevance and comprehensibility of the items, and thus the content validity considered strong. While further evaluations on the validity and reliability of the SILQ were performed in Sweden, such as through Rasch analysis, the development of the Dutch SILQ-NL37 began parallelly in collaboration between the research groups. Hence, these instruments share the same theoretical foundation but have been developed in slightly different directions, such as branches of the same tree, for instance due to differences in the Dutch and Swedish systems and culture. Results on the evaluations of the original SILQ are forthcoming.

The development process of the SILQ-NL37 included multiple steps. The aims of the adaptations were to ensure the measure represented the daily practice of the Dutch SSI, simplify language, and to shorten administration time. To reach the last aim, in step 1, one researcher selected the 3–5 items from the original SILQ that represented the Dutch SSI best. For example, a question regarding the understanding of leaflets was left out, because these were not used anymore as information was digitalized. In step 2, two other researchers and four employees of the SSI checked all items. They supported to simplify language, but also how items could be improved to meet daily practice within the Dutch SSI. In a last step, the adaptations were checked a second time by the same persons, and small changes in language were made until the SILQ-NL37 was considered final for our study.

The final SILQ-NL37 consisted of 37 items with 5-point disagreement-agreement Likert scales, where ‘strongly disagree’ gave a score of 1 on a specific item and ‘strongly agree’ a score of 5. Participants could also answer ‘not applicable/don’t know’. To explore the individual abilities and system comprehensibility, the total SILQ-NL37 scores were calculated for each participant by summing up the answers on 25 items on individual abilities and 12 items on system comprehensibility separately. The total scores were expressed as a percentage between 0 and 100 by dividing the calculated score by the total possible score (125 for individual abilities, and 62 for system comprehensibility), and multiplying by 100. Table 1 defines the measured abilities and domains within our study, and also shows how many items were asked within those abilities and domains.

**Table 1:** The SILQ-NL37 measured both individual abilities and system comprehensibility

	Individual abilities (25 items)			System comprehensibility (12 items)	
Domains ↓﻿	Obtaining information (8 items)	Understanding Information (11 items)	Acting upon information (6 items)	Domains ↓	
Contacts and communication with the system (8 items)	You can find information by communicating with the system	You can understand information from the system	You can provide information to and communicate with the system	Contacts and communication with the system (6 items)	Information is easily accessible, adequate and provided at the right time
Navigating the system (10 items)	You can find information about regulations and processes	You can understand regulations and processes in relation to your situation	You can navigate and act in the system	Navigating the system (3 items)	Staff in the system help you to understand and navigate the system
Decisions and appeals (7 items)	You can find information about decisions and how you can respond to them	You can understand reasons for decisions and their consequences	You know how to act after a decision	Decisions and appeals (3 items)	Decisions are predictable and transparent

The items regarding individual abilities focused on obtaining, understanding and acting upon information within different domains. The same domains were used for the items to measure system comprehensibility

*Socio-economic characteristics*—To describe the study sample and to examine associations of SIL with socio-economic characteristics, we received data on age, gender, education, postal code and living situation, already saved in the questionnaire system. In addition, the ability to understand and speak the Dutch language (Dutch language skills), and years of receiving a work disability benefit, were asked in the questionnaire. The SSI also provided additional information on age, gender and educational level for all approached panel members and the total Dutch population with work disability benefits, allowing for comparison of group distributions for these three characteristics with our study sample to estimate the representativeness of our participants.

### Statistical Analyses

All statistical analyses were performed in SPSS, version 26.0. From the respondents, we excluded participants with ≥ 15% ‘not applicable/don’t know’ answers from the analysis on total scores for both individual abilities and system comprehensibility. While for specific ability and domain analyses, only complete cases were included. To check the effects of this exclusion, chi-square tests were performed to compare the excluded and included participants, and to learn if group distributions among those participants were different. For age, education, Dutch language skills, years of work disability benefits and postal codes, we regrouped variables into three groups. From multiple answers on living situation, we extracted if a participant was with or without a partner. This was done to optimize group sizes for statistical analyses. Details on the original groups are in Online Appendix 1.

In addition to the evaluations of the SILQ in Sweden, we measured internal consistency–reliability to explore the measurement properties of the SILQ-NL37. We calculated the *Cronbach α* for the full set of items measuring individual SIL and system comprehensibility, but also for separate domains and abilities. Additionally, we used *Spearman correlation tests (rho)* to examine correlations between and within the different abilities and domains of the SIL. *Cronbach α* indicated high internal consistency-reliability for all items measuring individual SIL (0.93) and system comprehensibility (0.94). For the independent abilities and domains consistency-reliability was moderate to high (0.77–0.91). *Spearman rho’s* indicated a moderate to strong correlation between different abilities and domains of the SIL (rho = 0.54–0.80). We found weak to strong item-correlations within the independent abilities and domains of the SILQ-NL37. Online Appendix 2 and 3 give the results in detail.

Descriptives and frequencies were used to describe the means and standard deviations (SD) of the SILQ-NL37 scores for all participants and within subgroups, organized according to the collected socio-economic background characteristics.

To determine the prevalence of limited SIL, we used pairwise k-means clustering to create two groups of limited and adequate SIL, similar to HL studies [8, 17]. K-means clustering is a data driven method in which SPSS creates a predefined number of groups with similar answer patterns, determined by having as low variance as possible among responses on the different variables in each group. In our clustering procedure, we preset the number of groups on two. SPSS then ran 50 iterations with all 25 variables for individual SIL abilities. During an iteration and for each independent variable, SPSS created two groups with respondents as closely centered around an emerging mean. The grouping results of the independent variables were combined to divide people into the groups of limited and adequate SIL. After 50 iterations, this procedure led to two groups of limited and adequate SIL, with low within group variance. We used frequencies to describe group distributions among these groups.

To examine associations between SIL and socio-economic characteristics, we performed three analyses. Among participants with limited and adequate SIL, we performed Chi Square tests to detect significant differences in the two groups created with k-means clustering. Second, to examine associations between socio-economic factors and total SILQ-NL37 score, we calculated within group means for both the total individual abilities and system comprehensibility score. We performed independent t-tests for binary variables and linear regression analyses for categorical variables to find associations with the socio-economic characteristics. Third, to gain additional insight in associations between socio-economic characteristics and the SILQ-NL37 score, analyses were performed for the three separate subdomains; Contacts and Communication, System Navigation and Decisions and Appeals and for the three abilities; obtaining, understanding and acting upon information. We calculated the within group means and checked for significant group differences with independent t-tests and linear regression analyses for categorical variables. We performed *Bonferroni post-hoc tests* to correct for the multiple group comparisons in our analyses. Only results that remained significant after *Bonferroni post-hoc tests* were reported.

## Results

### Descriptives

Table 2 shows the socio-economic characteristics of the 567 (32%) study participants. About 55% was male and 74% was aged ≥ 50 years. Study participants closely represented the members of the online panel, but were more often male, older and higher educated compared to the total population receiving disability compensation (see Online Appendix 1). From the 567 respondents, 57 respondents were excluded from the analyses on total scores for individual abilities, and 91 from the analyses on total scores for system comprehensibility due to ≥ 15% ‘not applicable/don’t know’ answers. These respondents were lower educated and more often had bad to moderate language skills differed compared to the included sample. The mean SILQ-NL37 score for the sample was 71.2 (SD = 12.9) for individual abilities to obtain, understand and act on information of the social insurance system. The mean SILQ-NL37 score for system comprehensibility was 66.3 (SD = 15.8).

**Table 2:** Characteristics of the study participants

	Study participants N(%)
Total	567
Sex	
Male	305 (54.6)
Female	254 (45.4)
Age (in years)	
18–34	16 (3.1)
35–49	120 (23.3)
50–59	217 (42.2)
> 60	161 (31.3)
Education	
Low	134 (24.8)
Middle	215 (39.8)
High	191 (35.4)
Region	
Northeast	172 (30.9)
South	130 (23.3)
West	255 (45.8)
Partnered	
No	210 (38.8)
Yes	331 (61.2)
Years of illness compensation	
< 1 year	29 (5.1)
1–2 years	128 (22.6)
3–5 years	237 (41.8)
5–10 years	143 (25.2)
> 10 years	30 (5.3)
Ability to speak and understand Dutch	
Very good	430 (75.8)
Good	94 (16.6)
Sufficient	39 (6.9)
Moderate	2 (0.4)
Bad	2 (0.4)

N = number of participants. When data is missing, numbers do not sum up to 567

### Limited and Adequate SIL

Of all participants, 34.9% had limited SIL, which is characterized by a total individual SILQ-NL37 mean score of 57.4 (SD = 8.8). 65.9% of the participants had adequate SIL, which is characterized by a mean score of 78.6 (SD = 7.4). Participants in the group with limited SIL, significantly more often were without a partner (p = 0.018). Participants with limited SIL generally disagreed or neither agreed or disagreed with the items measuring obtaining, understanding and acting upon information within the different domains, meaning that they had doubts about possessing the needed abilities (mean item scores: 2.34–3.46). Participants with adequate SIL, generally, agreed or strongly agreed (mean item scores: 3.45–4.29) they possessed the needed abilities to obtain, understand and act on information within the different domains. Table 3 gives more information on group distributions.

**Table 3:** Group distributions of people with adequate and limited social insurance literacy and within group total mean SILQ-NL37 scores for both individual abilities and system comprehensibility with the p-values from statistical tests

	Individual abilities						System comprehensibility		
	Adequate social insurance literacy	Limited social insurance literacy		Total score			Total score		
	N (%)	N (%)	p	N	Mean(SD ± range)	p	N	Mean(SD ± range)	p
Total	332 (65.1)	178 (34.9)		510	71.2 (12.9 ± 75.2)		475	66.3 (15.8 ± 80.0)	
Sex									
Male	174 (53)	103 (59.2)	0.220	277	70.4 (13.4 ± 75.2)	0.119	261	66.1 (16.5 ± 80.0)	0.641
Female	154 (47)	71 (40.8)		225	72.2 (12.0 ± 68.0)		207	66.8 (15.0 ± 80.0)	
Age (in years)									
18–49	82 (27.3)	41 (25.2)	0.260	123	71.6 (13.2 ± 68.0)	*0.081*	116	64.2 (16.9 ± 80.0)	0.101
50–59	115 (38.3)	75 (46.0)		190	*69.6 (13.5* ± *75.2)*		181	65.8 (15.3 ± 80.0)	
> 60	103 (34.3)	47 (38.8)		150	72.7 (11.7 ± 57.6)		133	68.4 (15.7 ± 73.3)	
Education									
Low	71 (22)	41 (25.0)	0.635	112	71.0 (11.5 ± 50.4)	0.691	104	68.8 (14.3 ± 72.0)	0.146
Middle	127 (39.3)	66 (40.2)		193	71.1 (14.4 ± 75.2)		189	67.0 (16.9 ± 80.0)	
High	125 (38.7)	57 (34.8)		182	72.1 (11.9 ± 60.8)		162	65.0 (15.2 ± 73.3)	
Region									
Northeast	103 (31.5)	48 (27.7)	0.577	**151**	73.1 (12.4 ± 75.2)*	**0.031**	138	67.8 (15.4 ± 80.0)	0.438
South	72 (22.0)	44 (25.4)		**116**	68.9 (13.3 ± 64.3)*		111	65.5 (14.8 ± 73.3)	
West	152 (46.5)	81 (46.8)		233	71.2 (12.5 ± 68.2)		217	66.0 (16.4 ± 73.3)	
Partnered									
No	**111 (34.8)**	**77 (46.1)**	**0.018**	188	*69.9 (13.6* ± *75.2)*	*0.064*	176	*64.6 (17.4* ± *80.0)*	*0.085*
Yes	**208 (65.2)**	**90 (53.9)**		298	*72.1 (12.4* ± *70.4)*		275	*67.3 (15.1* ± *80.0)*	
Years of illness compensation									
0–2 years	93 (28.0)	50 (28.1)	0.836	143	71.1 (13.8 ± 75.2)	0.484	136	67.3 (17.1 ± 80.0)	0.214
3–5 years	144 (43.4)	73 (41.0)		217	71.9 (12.1 ± 62.5)		204	67.0 (15.1 ± 73.3)	
> 5 years	95 (28.6)	55 (30.9)		150	70.3 (13.0 ± 68.0)		135	64.3 (15.5 ± 80.0)	
Ability to speak and understand Dutch									
Very good	267 (80.4)	133 (74.7)	0.265	400	71.8 (13.2 ± 75.2)	0.141	370	*65.4 (16.3* ± *80.0)*	*0.063*
Good	47 (14.2)	30 (16.9)		77	69.8 (10.8 ± 57.1)		72	*69.0 (14.6* ± *72.0)*	
Sufficient to bad	18 (5.4)	15 (8.4)		53	67.9 (12.4 ± 55.0)		33	*70.5 (11.9* ± *66.3)*	

Bold numerals show a significant result (P < 0.05). Italic numerals show a result close to significance (< 0.1)

* = the significantly different group. The result for living region remained significant after Bonferroni post-hoc tests in SPSS (p = 0.025)

N = number of participants. SD = standard deviation. p = p-value

The mean total individual abilities score was derived from answers on 25 items of the SILQ-NL37

The mean total system comprehensibility score was derived from 12 items of the SILQ-NL37. A total of N = 57 (10%) respondents for the analyses on individual abilities and N = 93 (16%) respondents for analyses on system comprehensibility were excluded

According to our group comparisons, the group of excluded participants had significantly higher proportions of lower educated people and people with bad to sufficient language skills.’

### Individual SIL and Socio-economic Characteristics

Several findings revealed an association between individual SIL scores and socio-economic characteristics. A higher individual SILQ-NL37 total score was significantly associated with living in the northeastern region of the Netherlands (p = 0.031) compared to the southern region. Additionally, though non-significant, the analysis indicated a lower individual SILQ-NL37 total score was associated with being between 50 and 59 years compared to being over 60 years (p = 0.081) and being without a partner, compared to being with a partner (p = 0.064). Table 3 gives results on the relationship between individual SIL scores and socio-economic characteristics.

Additionally, there were several associations between SIL abilities (obtaining, understanding and acting upon information) and domains (contacts and communication, navigating the system and decisions and appeal), and socio-economic characteristics. A lower mean score for the ability to obtain (p = 0.041) and understand information (p = 0.049) was significantly associated with being of male sex, in comparison with being of female sex. A lower mean score for the ability to act on information was significantly associated with being between 50–59 years, compared to the younger age group (p = 0.020). A lower mean score for the ability to act upon information was associated with having bad to sufficient Dutch language skills, compared to having good (p = 0.043) and very good Dutch skills (p =  < 0.001). A higher mean score on the domain contacts and communication was significantly associated with living in the northeastern region, compared to living in the southern region (p = 0.002). A lower mean score on the domain decisions and appeal compared was significantly associated with being without a partner, in comparison to being with a partner (p = 0.008). Details are in Table 4.

**Table 4:** Group means for the different individual abilities and for all items within obtaining, understanding and acting on information and for all items within the subdomains Contacts and Communication, System Navigation and Decisions and Appeals, administered with the SILQ-NL37

	Obtaining information		Understanding information		Acting on information		Contacts and Communication		System Navigation		Decisions and Appeals	
	N	Mean(SD)	N	Mean(SD)	N	Mean(SD)	N	Mean(SD)	N	Mean(SD)	N	Mean(SD)
Sex												
Male	**202**	**3.31 (0.75)**	**228**	**3.46 (0.71)**	233	3.65 (0.66)	260	3.68 (0.71)	222	3.47(0.75)	211	3.42 (0.68)
Female	**162**	**3.46 (0.66)**	**185**	**3.60 (0.71)**	190	3.68 (0.68)	209	3.78 (0.63)	170	3.57 (0.71)	178	3.49 (0.68)
Age (in years)												
18–49	86	3.40 (0.71)	103	3.59 (0.71)	**108**	**3.76 (0.70)**	117	3.78 (0.67)	92	3.51 (0.78)	94	3.56 (0.72)
50–59	139	3.32 (0.70)	154	3.45 (0.75)	**159**	**3.54 (0.66)**	177	3.66 (0.71)	150	3.46 (0.76)	155	3.38 (0.65)
> 60	111	3.47 (0.74)	123	3.55 (0.66)	128	3.71 (0.64)	139	3.76 (0.66)	118	3.60 (0.66)	114	3.46 (0.70)
Education												
Low	88	3.50 (0.72)	99	3.55 (0.62)	92	3.58 (0.56)	104	3.69 (0.63)	94	3.63 (0.64)	92	3.40 (0.77)
Middle	146	3.37 (0.71)	163	3.55 (0.78)	164	3.64 (0.75)	188	3.75 (0.69)	159	3.53 (0.81)	150	3.46 (0.74)
High	117	3.33 (0.70)	141	3.50 (0.70)	154	3.76 (0.63)	164	3.74 (0.69)	126	3.45 (0.69)	135	3.51 (0.61)
Region												
Northeast	96	3.41 (0.68)	121	3.62 (0.66)	124	3.77 (0.60)	**145**	**3.86 (0.64)**	107	3.59 (0.71)	113	3.51 (0.60)
South	89	3.33 (0.68)	96	3.40(0.73)	102	3.57 (0.70)	**104**	**3.56 (0.66)**	101	3.50 (0.75)	93	3.33 (0.76)
West	177	3.39 (0.73)	194	3.53 (0.71)	195	3.64 (0.68)	218	3.72 (0.68)	182	3.48 (0.71)	181	3.47 (0.67)
Partnered												
No	133	3.33 (0.76)	148	3.45 (0.76)	160	3.58 (0.71)	179	3.72 (0.69)	138	3.44 (0.75)	**141**	**3.33 (0.69)**
Yes	219	3.40 (0.69)	251	3.56 (0.70)	250	3.71 (0.64)	274	3.73 (0.67)	241	3.55 (0.73)	**235**	**3.52 (0.67)**
Dutch ability												
Very good	282	3.35 (0.73)	321	3.53 (0.74)	**339**	**3.73 (0.66)**	372	3.76 (0.68)	303	3.53 (0.75)	302	3.49 (0.68)
Good	59	3.42 (0.62)	69	3.51 (0.58)	**64**	**3.51 (0.52)**	71	3.62 (0.64)	67	3.52 (0.63)	67	3.31 (0.58)
Sufficient to bad	29	3.53 (0.64)	30	3.43 (0.74)	**27**	**3.20 (0.82)**	32	3.57 (0.67)	28	3.31 (0.75)	27	3.23 (0.73)
Years of compensation												
0–2 years	101	3.46 (0.70)	115	3.51 (0.74)	117	3.64 (0.75)	131	3.72 (0.68)	112	3.56 (0.78)	112	3.46 (0.72)
3–5 years	164	3.37 (0.68)	182	3.58 (0.67)	185	3.69 (0.61)	206	3.77 (0.64)	173	3.53 (0.67)	162	3.40 (0.64)
> 5 years	105	3.31 (0.76)	123	3.44 (0.75)	128	3.64 (0.67)	138	3.67 (0.72)	113	3.42 (0.76)	122	3.50 (0.68)

Bold numerals show a significant different result (P < 0.05). Only results that remained significant after Bonferroni post-hoc analyses were reported. N = number of participants in each group in each analysis. N-differences are explained by the exclusion of participants with ‘not applicable/don’t know’ answers, since we only included respondents who answered all items within the studied domain or ability in our analyses. SD = standard deviation, ^#^ According to our group comparisons, the group of excluded participants had significantly higher proportions of lower educated and people with bad to sufficient language skills

### System Comprehensibility and Socio-economic Characteristics

No significant associations between total system comprehensibility SILQ-NL37 total score and socio-economic characteristics were found (see Table 3). However, the analysis indicated, though non-significant, a higher reported score for system comprehensibility was associated with having bad to sufficient Dutch language skills (p = 0.063), and being of lower education (p = 0.146) or with a partner (p = 0.085), compared to people with better skills, higher education or without a partner.

There were a few associations between specific domains of system comprehensibility and socio-economic characteristics. Lower mean scores for the domains navigating the system (p = 0.009) and decisions and appeal (p = 0.018) were associated with being between 18–49 years, compared to being of older age. A lower mean score for the domain navigating the system also was associated with having high education (p = 0.026), very good ability to speak and understand Dutch (p = 0.008), and being without a partner (p = 0.016), compared to having low education and bad to sufficient Dutch ability or being with a partner. Details are in Table 5.

**Table 5:** Group means for system comprehensibility for all items within the subdomains Contacts and Communication, System Navigation and Decisions and Appeals, administered with the SILQ-NL37

	Contacts and Communication		System Navigation		Decisions and Appeals	
	N	Mean(SD)	N	Mean(SD)	N	Mean(SD)
Sex						
Male	241	3.34 (0.78)	241	3.22 (1.05)	251	3.23 (0.98)
Female	192	3.39 (0.74)	201	3.18 (0.95)	202	3.33 (0.83)
Age (in years)						
18–49	109	3.30 (0.85)	108	**2.96 (1.06)**	**110**	**3.08 (0.96)**
50–59	163	3.33 (0.72)	169	3.20 (1.01)	179	3.24 (0.90)
> 60	122	3.42 (0.74)	127	**3.36 (0.96)**	**130**	**3.41 (0.89)**
Education						
Low	92	3.44 (0.67)	**96**	**3.43 (0.92)**	104	3.44 (0.82)
Middle	172	3.42 (0.83)	182	3.23 (1.03)	176	3.21 (0.97)
High	156	3.28 (0.72)	**151**	**3.08 (1.02)**	161	3.29 (0.87)
Region						
Northeast	126	3.45 (0.74)	137	3.27 (0.96)	137	3.36 (0.88)
South	103	3.35 (0.72)	98	3.19 (0.98)	107	3.16 (0.92)
West	202	3.31 (0.78)	206	3.18 (1.04)	207	3.28 (0.93)
Partnered						
No	162	3.27 (0.84)	**167**	**3.05 (1.07)**	173	3.18 (0.98)
Yes	256	3.40 (0.72)	**259**	**3.29 (0.97)**	263	3.32 (0.89)
Dutch ability						
Very good	352	3.32 (0.79)	**344**	**3.15 (1.03)**	354	3.22 (0.94)
Good	63	3.48 (0.69)	72	3.33 (0.95)	72	3.44 (0.79)
Sufficient to bad	25	3.44 (0.57)	**32**	**3.58 (0.72)**	34	3.45 (0.83)
Years of compensation						3.39 (0.98)
0–2 years	120	3.40 (0.84)	130	3.25 (1.05)	134	
3–5 years	189	3.36 (0.74)	189	3.28 (0.96)	193	3.27 (0.84)
> 5 years	131	3.30 (0.73)	129	3.06 (1.00)	133	3.16 (0.95)

Bold numerals show a significant different result (P < 0.05). Only results that remained significant after Bonferroni post-hoc analyses were reported. N = number of participants in each group in each analysis. N-differences are explained by the exclusion of participants with ‘not applicable/don’t know’ answers, since we only included respondents who answered all items within a domain in our analysis. SD = standard deviation. ^#^ According to our group comparisons, the group of excluded participants had significantly higher proportions of lower educated and people with bad to sufficient language skills

## Discussion

Our study shows that limited social insurance literacy is a prevalent problem (35%) in Dutch people receiving work disability benefits. Higher individual total SIL scores and scores for specific abilities and domains of the SILQ-NL37 are associated with several socio-economic characteristics, for example having a partner, northern living region, better Dutch language skills, and an age between 50 and 59 years. People with a partner, lower education and limited Dutch language skills consider the social insurance system more comprehensible.

The prevalence of limited SIL is in line with studies on health literacy (HL) in the Netherlands. In a comparative study on HL across Europe, 29% of the Dutch respondents had limited HL, while the mean for all participating countries was 48% [18]. In this study, the European Health Literacy Survey was used, which contains domains that are similar with the SILQ, such as accessing and understanding health care information and applying information to improve health. The similar prevalence rates for SIL and HL give an indication that the SILQ is able to identify people with problems to obtain, understand and act upon information, which may influence social insurance related outcomes, such as perceived system justice or the awarding of benefits. Based on the HL study [18], we expect that country-related differences exist for SIL as well, which needs further exploration in research.

The identified associations between SIL and socio-economic factors are comparable to associations of related concepts. Our study shows lower individual total and within domain SILQ-NL37 mean scores are associated with male sex, limited Dutch language skills, southern living region and higher age. These results largely confirm the results of studies on HL and financial literacy [12, 18–20]. The retrieved association between individual score and ‘not having a partner’ may indicate that SIL is more dependent on an interplay of factors within the direct social context. This is different from studies on HL, where associations of ‘being non-married’ with HL give inconsistent results [21]. A possible explanation is that individuals need more support from others when navigating through SSIs, compared to patients in health organizations. This is potentially a result of encountering multiple negative life events at once, since these people are both ill and fear to become unemployed at the same time. Another explanation is that processes are more complex than in health organizations, because of strict and bureaucratic regulations and the involvement of multiple stakeholders, from the employer, SSI and health organizations. Other studies have indicated the importance of emotional and practical support of the social network, but also the employer, to deal with diseases and to navigate through social insurance processes [13, 14].

The higher SILQ-NL37 mean score of people living in the northeastern region, might be related to differences between regions in income, type of work, and familial background [11, 22]. These factors might act as confounders, which could also be the case for the other socio-economic factors in our study, for which we did not correct. The fact that in the northeastern region of the Netherlands, unemployment rates are higher and incomes lower [23], is giving an indication confounding could play a role. Based on this, a possible explanation for the higher SIL in this region is that inhabitants may have more experience with social insurance systems, and that they hence have learned how to interact with the SSI. Another explanation could be that employers and local governments provide better support compared to the offered support in other regions. Although, the true explanation remains uncertain, and more insight in associations between SIL, living area and other socio-economic factors is necessary to unravel the precise role of these factors in social insurance systems.

The finding that people with lower education and limited Dutch language skills rate the system as more comprehensive, appears counterintuitive. There are several explanations possible. It could be that the participants only think they understand the system, because they are unable to comprehend all the details. Another explanation is that these people tend to blame themselves for not understanding the system, instead of critically reflecting on the system comprehensibility. There is limited evidence on these kind of relationships, but a finding that patients with less knowledge [24] and lower HL [25] were more satisfied and less critical towards the communication of the health care professional, indicates that understanding is a factor that might influence the rating of system performance. Although other studies indicate the opposite [26]. A more positive interpretation is that the system communicates better with, or organizes additional help for, people with lower education or less Dutch language skills. If this interpretation is correct, it suggests that the system is successful in differentiating according to individual skills. Qualitative studies could play a role in unraveling the reasons behind the finding that people with less education and language proficiency rate the system as more comprehensible.

### Strengths and Limitations

Our study has strengths and limitations. A first strength is that we approached SIL in various ways by using a clustering method, total individual and system SILQ-NL37 scores and scores for specific abilities and within domains. We thereby provide the first comprehensive overview of the role of limited SIL in social insurance systems and its association with socio-economic characteristics. A second strength is that we, with help of employees of the SSI, adapted the preliminary SILQ to the Dutch context. With this approach, we ensured the questionnaire closely reflected the Dutch social insurance system, which prevents information bias as a result of administering unsuitable questions.

The most important limitation refers to a high risk of selection bias on several levels. First, the recruitment via the online panel leads to bias, as members have pro-actively signed up for an online panel and need skills to do that. Compared to the general population, participants were higher educated (53% vs. 25%). Most had good to very good Dutch skills (91%), which is expectedly better than in general, as we know around 25% of the people with disability benefits are migrants. It is possible the included participants with low education in our study are not representative for clients of the SSI with low education in general, for example because they have better skills in reading or using digital technology. This offers a potential explanation why we don’t find an association between individual SIL and educational level. Second, in all analyses, we excluded participants with ‘not applicable / don’t know’ answers. Our group comparisons indicated the excluded participants were lower educated and more often had bad to moderate language skills. This limits the possibility to generalize our results. Based on the above, we expect to underestimate the prevalence and role of limited SIL. Third, our participation rate of 32% is too low according to study quality assessment instruments [27]. However, since the participants closely represented all online panel members, we expect that participation bias did not influence the results. Another limitation is that our *spearman correlation* tests did not always show strong correlations, and this might result in measurement bias. Although we cannot be sure. If we assume, for example, obtaining, understanding and acting upon information are similar abilities, strong correlations are expected. But, if we treat them as different individual non-related abilities, it is possible correlations are moderate or even low. This uncertain measurement validity might also have influenced the results of our study. So in this case, the upcoming validation study of the original SILQ might add to the evidence and provide answers.

### Implications

Our study has implications for research and practice. More research is essential to further unravel the role of SIL in social insurance systems. Since SIL is a new concept, it is important to validate the original SILQ, which is currently undertaken by Swedish researchers. Larger cross-country cohort-studies can help to gain insight in the prevalence of limited SIL, associations of SIL with socio-economic characteristics, but also if these characteristics act as confounders. Based on our results, we suggest to further unravel the role of social support, as a potential proxy measure of SIL. Additionally, these studies should unravel the influence of SIL on different social insurance related outcomes, such as received work disability benefits and perceived system justice. This is important because we know limited HL has a negative impact on multiple outcomes, such as active participation in the treatment [28] and mobility during rehabilitation from physical injuries [29, 30]. Additionally, it is important to learn more about the role of professionals in their contact with people with limited SIL, for example if they are able to tailor their communication, and to develop and test interventions targeting clients and professionals in social insurance systems to optimize support of people with limited SIL.

This study also has implications for practice, especially since the number of people receiving work disability benefits has risen in multiple countries [31, 32]. Our study shows a group of 35% with limited SIL expresses to have lacking abilities to obtain, understand and act upon information. We therefore suggest social security institutes to take action to identify people with limited SIL and support these people better. Based on HL research [33], we advise to simplify written information and use more visual strategies. Another approach would be to make employees more aware of limited SIL and train them in strategies that were effective for people with limited HL, such as providing easy information, checking understanding and including the social network [34].

## Conclusion

Our study shows a group of 35% experiences limited SIL and thereby expresses to have lacking abilities to obtain, understand and act upon information. Lower total SILQ-NL37 scores and scores for specific abilities and within domains are associated with socio-economic background factors, such as not having a partner, limited ability to speak Dutch, and living area. Counterintuitively, people with low education and bad to sufficient ability to speak and understand Dutch rate the system comprehensibility higher. To broaden the knowledge on the influence of SIL on navigating social insurance processes or applying for and granting of benefits, future researches should study the concept in a more representative sample, and in different countries and social insurance contexts.

## Supplementary Information

Below is the link to the electronic supplementary material.

Supplementary file1 (DOCX 21 kb)

Supplementary file2 (DOCX 16 kb)

Supplementary file3 (DOCX 111 kb)

## Data Availability

Data and material are available on request due to privacy or other restrictions.
