# Supplementary material for: Social Insurance Literacy of Dutch Workers Receiving Disability Benefits and its Associations with Socio-Economic Characteristics
Source: J Occup Rehabil. 2022 Jan 5;32(3):494–504. doi: 10.1007/s10926-021-10018-3 (PMC9576638; doi:10.1007/s10926-021-10018-3)
Supplement: Supplementary file 1 — Supplementary file1 (DOCX 21 kb) [file 10926_2021_10018_MOESM1_ESM.docx]

**Appendix 1.** Characteristics of people with illness compensation, based on the regulations of the Dutch Labour Capacity Act for the total population, all members of the online panel and study participants

|  | Total population^#^  N(%) | All panel members^~^ N(%) | Study participants N(%) |
| --- | --- | --- | --- |
| Total | 199.436 | 1753 | 567 |
| Sex |  |  |  |
| Male | 90.284 (45.3) | 859 (49.0) | 305 (54.6) |
| Female | 109.150 (54.7) | 894 (51.0) | 254 (45.4) |
| Age (in years) |  |  |  |
| 18-34 | 19.863 (10.0) | 70 (4.0) | 16 (3.1) |
| 35-49 | 72.058 (36.1) | 491 (28.0) | 120 (23.3) |
| 50-59 | 66.060 (33.1) | 666 (38.0) | 217 (42.2 ) |
| > 60 | 41.455 (20.8) | 508 (29.0) | 161 (31.3) |
| Education |  |  |  |
| Low | 84.277 (53.0) | 368 (21.0) | 134 (24.8) |
| Middle | 49.719 (31.3) | 736 (43.5) | 215 (39.8) |
| High | 25.065 (15.8) | 526 (30.0) | 191 (35.4) |
| Region |  |  |  |
| North |  |  | 56(10.1) |
| South |  |  | 130(23.3) |
| East |  |  | 116(20.8) |
| West |  |  | 255(45.8) |
| Partnered |  |  |  |
| No |  |  | 210 (38.8) |
| Yes |  |  | 331(61.2) |
| Years of illness compensation* |  |  |  |
| <1 year | 27.683 (13.9) |  | 29 (5.1) |
| 1-2 years | 46.575 (23.3) |  | 128 (22.6) |
| 3-5 years | 47.965 (24.1) |  | 237 (41.8) |
| 5-10 years | 56.987 (28.6) |  | 143 (25.2) |
| >10 years | 20.226 (10.1) |  | 30 (5.3) |
| Ability to speak and understand Dutch* | | | |
| Very good |  |  | 430 (75.8) |
| Good |  |  | 94 (16.6) |
| Sufficient |  |  | 39 (6.9) |
| Moderate |  |  | 2 (0.4) |
| Bad |  |  | 2 (0.4) |

N= number of participants; when data is missing numbers do not sum up to 567. ^#^ Originating from the Center of Central Expertise, Department of Knowledge and Innovation, of the Dutch Employee Insurance Agency, reference date: 30 November 2019. ^~^ Originating from the Department of Client and Service of the Dutch Employee Insurance Agency, reference date: 30 November 2019. * For these variables, data were not collected for the total population or all panel members by the references of the data.
