# Supplementary material for: Social Insurance Literacy of Dutch Workers Receiving Disability Benefits and its Associations with Socio-Economic Characteristics
Source: J Occup Rehabil. 2022 Jan 5;32(3):494–504. doi: 10.1007/s10926-021-10018-3 (PMC9576638; doi:10.1007/s10926-021-10018-3)
Supplement: Supplementary file 2 — Supplementary file2 (DOCX 16 kb) [file 10926_2021_10018_MOESM2_ESM.docx]

**Appendix 2.** Internal consistency-reliability (Cronbach’s alpha) and within ability and domain item correlations (Spearman rho) of the SILQ-NL37.

| **Individual abilities (25 items)** | | | |
| --- | --- | --- | --- |
| **Competency domain** | **No. of items** | **Spearman rho** | **Cronbach’s alpha** |
| Obtaining information | 8 items | 0.229-0.725 | 0.834 |
| Understanding information | 11 items | 0.245-0.748 | 0.880 |
| Acting on information | 6 items | 0.230-0.622 | 0.770 |
| **System Domain** | **No. of items** | **Spearman rho** | **Cronbach’s alpha** |
| Contacts and Communication | 8 items | 0.279-0.725 | 0.851 |
| Navigation of the System | 10 items | 0.243-0.748 | 0.899 |
| Decisions and Appeals | 7 items | 0.261-0.597 | 0.795 |
| **System comprehensibility (12 items)** | | |  |
| **System Domain** | **No. of items** | **Spearman rho** | **Cronbach’s alpha** |
| Contacts and Communication | 6 items | 0.354 – 0.618 | 0.855 |
| Navigation of the System | 3 items | 0.738 – 0.808 | 0.912 |
| Decisions and Appeals | 3 items | 0.468 – 0.641 | 0.802 |
