# Supplementary material for: Social Insurance Literacy of Dutch Workers Receiving Disability Benefits and its Associations with Socio-Economic Characteristics
Source: J Occup Rehabil. 2022 Jan 5;32(3):494–504. doi: 10.1007/s10926-021-10018-3 (PMC9576638; doi:10.1007/s10926-021-10018-3)
Supplement: Supplementary file 3 — Supplementary file3 (DOCX 111 kb) [file 10926_2021_10018_MOESM3_ESM.docx]

**Appendix 3.** Correlation between different abilities and domains of the SILQ-NL37 for both individual abilities and system comprehensibility

**
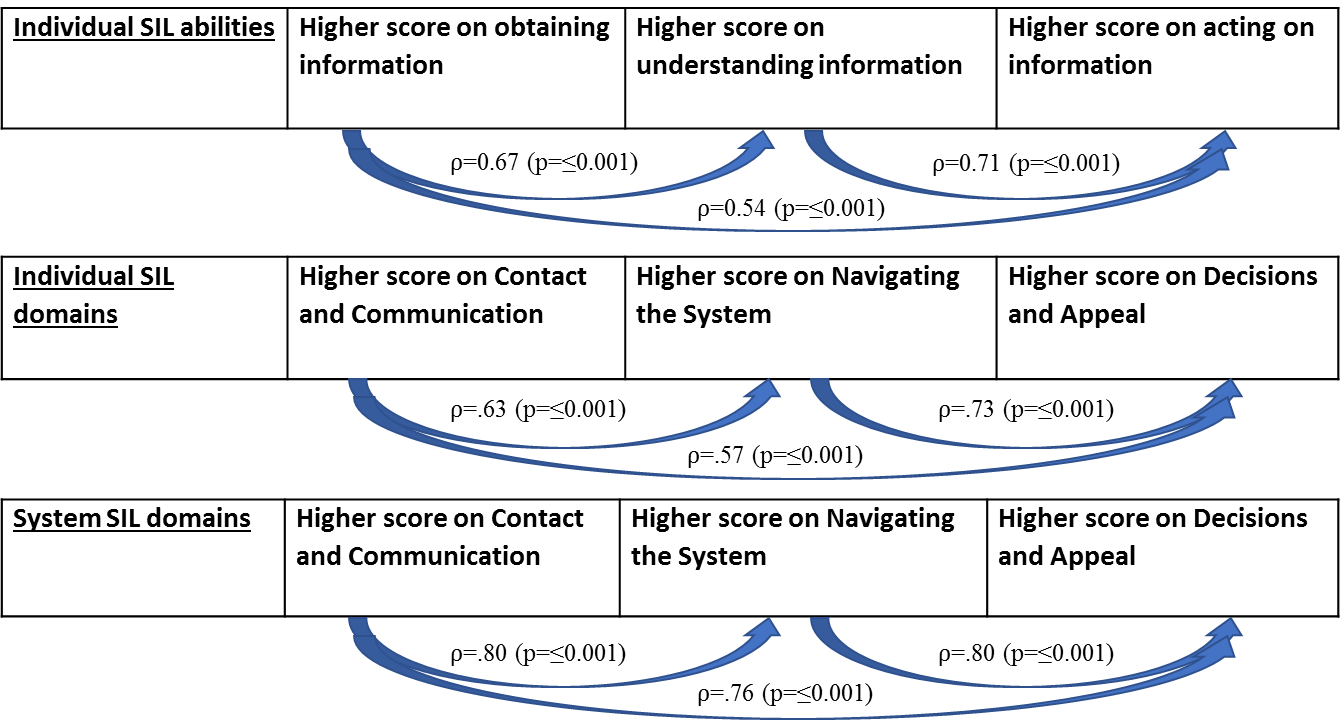
**

*ρ = spearman rho, indicating the strength of a correlation between two different domains*

*p = p-value*
